# Cyberattacks Defense in Digital Music Streaming Platforms by Mobile Distributed Machine Learning

**DOI:** 10.1155/2022/1701266

**Published:** 2022-05-06

**Authors:** Guoxu Fan

**Affiliations:** Pingdingshan University, Conservatory of Music, Pingdingshan, Henan 467000, China

## Abstract

Given the massive popularity of digital music industry repositories and their corresponding targeting by cybercriminals, this paper presents an intelligent model for cyberattacks defense in digital music streaming platforms by mobile distributed machine learning. The basic idea of machine learning is to use large data sets to create a model that responds well to inputs it has never processed before. With the increase in data volume and complexity of models, it becomes increasingly challenging to complete machine learning processes in a single machine. Distributed ML was developed to solve this problem, and a standard procedure is completed through the collaboration of multiple servers. With the evolution of mobile devices and the increase in their number, it is possible to create an integrated and compact mobile distributed machine learning (MDML) system that could reduce the workload of servers. A distributed logit polynomial function model is proposed, which is used to model options in distributed binary regression accounting units, which are of low complexity and high stability in noisy environments.

## 1. Introduction

One of the current challenges associated with the pandemic is the massive increase in content consumption through pay-TV and other streaming services. Users are forced to isolate themselves and have trapped many people at home [1]. From the first day of the enforcement of the measures, the music platforms available on the market and platforms for streaming content saw an increase in the number of their subscribers and the content they consume. For this reason, international streaming service providers have made recommendations for the need to reduce data rates to ensure the proper operation of networks, as the requirements have been maximized [2].

At the same time, cybercrime increased dramatically, especially the account takeover attack (ATA) [3] against music streaming platforms. Specifically, ATAs are a specialized type of attack in which criminals take ownership of online accounts using stolen access criteria to similar services. The basic techniques of these attacks are social engineering, brute force data breaches, and phishing campaigns. Botnets usually use the results of collecting these data to identify any other services that may use the same standards. In many cases, lists with similar data are sold on the dark web [4].

To further address these cases of breach and interception of credentials in a music industry service, this study proposes a highly efficient and easy-to-use MDML [5] that uses a polynomial logit function to model options in distributed binary regression units to timely detect cyberattacks in music content streaming services [6].

## 2. Related Literature

Mobile distributed machine learning and distributed computing [7] are concepts that the research community is currently trying to exploit in the best possible way the resources offered in each scenario [8, 9].

In 2015, Taddy [10] proposed a prototype method for the idea of distributed multinomial regression. Their research was fueled by the use of high-dimensional response multinomial models to analyze a large number of random counts. Content research, as texts are tokenized, and token counts are described as generating from a multinomial depending on document properties, was one of its inspiring uses. They developed such algorithms using text projected onto a broad collection of explanatory factors using a publicly accessible data set of Yelp reviews. The fitted models may investigate the relationship among phrases and variables of concern, reduce dimensions into supervised component scores, and predict outcomes. We suggest that the technique presented here is an appealing choice for social engineers and other textual researchers who want to use regression tools they are acquainted with on text data.

Shamili et al. [11] developed a dispersed support vector machine technique for detecting computer viruses on a system of portable devices in the context of network security. The lightweight design uses a statistical classification system developed via training with instances of both regular and exceptional use patterns to monitor cellular user behavior in a dispersed and privacy-preserving manner. They claim that the distributed learning technique has many benefits, including being lightweight regarding Internet use, maintaining the privacy of participating users, and automatically generating a generic behavioral signature of virus based on typical user usage patterns. The system was tested employing the MIT reality data set, and the results were positive.

Gu et al. [12] conducted a literature study on the server-based to client-based machine learning transition. They again went through a few popular server-based and client-based deep learning methodologies and applications. They also spoke about the obstacles and potential future developments in this field. They have described their goals and showed how client-based machine learning is both sufficient and necessary. They have highlighted the limitations of client-based inference and illustrated recent achievements, particularly in the disciplines of machine vision and natural language interpretation. Finally, they have identified future research paths in academia and business for client-based machine learning. In conclusion, implementing client-based machine learning in real-world applications is still in progress.

Shakarami et al. [13] presented a study of ML-based computation offloading techniques in the mobile edge computing ecosystem in the shape of a classical taxonomy to identify current mechanisms and unresolved concerns in this critical field. Reinforcement training, guided learning, and uncontrolled learning were the three primary categories in their proposed taxonomy. Then, depending on various characteristics, the applicable methodologies were compared to one another. Finally, they discussed several crucial research problems as outstanding topics in the ML-based offloading mechanisms, considering the existing literature gap.

In a cognitive eavesdropping context, Guo et al. [5] examined a distributed machine learning strategy for a multiuser mobile edge computing system in a perceptual eavesdropping context, where several secondary devices have specific tasks to compute with varied priorities. They looked at the federated learning methodology for the system architecture of a multiuser mobile edge computing platform. Various users had distinct computational tasks that needed to be calculated by different computational access points. Finally, simulation results showed that the suggested strategy might successfully minimize system costs in both delay and energy usage while also ensuring that the user with the highest job priority receives greater bandwidth and processing capabilities. In future research expanding from this study, they will investigate some fairness disparities between consumers due to aspects such as the network state, job size, and processing capabilities. Furthermore, since most mobile devices have a limited battery life, it is difficult to maintain all mobile devices online.

## 3. Methodology

The proposed implementation, based on distributed multinomial logistic regression (DMLR) [10], is a classification method that extends the capabilities to multiclass issues, such as those with more than two possible unique outcomes [12]. In statistics, it is a model used to forecast the probability of several possible effects of a categorically distributed dependent variable, given a set of independent variables that might have varied values such as real, binary, category, and other types [5, 14].

The model follows the same technique as the accounting regression, with the sole variation being that the dependent variables are categorical rather than binary. In particular, there are *K* alternative outcomes rather than just two. The proposed solution uses a linear prediction function *f*(*k, i*) to predict the probability that the observation *i* will have an effect *k*, of the following form [8, 15]:

(1) $$\begin{array}{c} f\left(k,i\right)=\beta_{0,k}+\beta_{1,k}x_{1,i}+\beta_{2,k}x_{2,i}+\cdots +\beta_{M,k}x_{M,i}, \end{array}$$

where *β*_*m*,*k*_ is a regression coefficient associated with the *m*-th explanatory variable and the *k*-th effect. Explanatory variables and coefficients are organized into vectors of magnitude *M* + 1 so that the prediction function can be written in its most elaborate form [5]:

(2) $$\begin{array}{c} f\left(k,i\right)=\beta_{k}\cdot x_{i}, \end{array}$$

where *β*_*k*_ is the set of coefficients related to the result *k*, and *x*_*i*_ (vector line) is the set of explanatory variables related to the observation *i*.

To arrive at the polynomial logit model, we use the logic that by running *K* − 1 independent regression accounting models, one result is selected as constant, and the other *K* − 1 results are regressed against the fixed result. If the result *K* (the last result) is chosen as the constant, then [5, 10]

(3) $$\begin{array}{c} \ln\frac{\mathrm{Pr}\left(Y_{i}=1\right)}{\mathrm{Pr}\left(Y_{i}=K\right)}=\beta_{1}\cdot {\text{X}}_{i},\\ \ln\frac{\mathrm{Pr}\left(Y_{i}=2\right)}{\mathrm{Pr}\left(Y_{i}=K\right)}=\beta_{2}\cdot {\text{X}}_{i},\\ \ln\frac{\mathrm{Pr}\left(Y_{i}=K-1\right)}{\mathrm{Pr}\left(Y_{i}=K\right)}=\beta_{K-1}\cdot {\text{X}}_{i}. \end{array}$$

For this reason, we defined different sets of regression constants, one for each possible result. Raising the power of *e* on both sides of each equation and solving in terms of probabilities, we have [16, 17]

(4) $$\begin{array}{c} \mathrm{Pr}\left(Y_{i}=1\right)=\mathrm{Pr}\left(Y_{i}=K\right)e^{\beta_{1}\cdot X_{i}},\\ \mathrm{Pr}\left(Y_{i}=2\right)=\mathrm{Pr}\left(Y_{i}=K\right)e^{\beta_{2}\cdot X_{i}},\\ \cdots \cdots \\ \mathrm{Pr}\left(Y_{i}=K-1\right)=\mathrm{Pr}\left(Y_{i}=K\right)e^{\beta_{K-1}\cdot x_{i}}. \end{array}$$

Using the fact that the sum of all *K* probabilities must make 1, then

(5) $$\begin{array}{c} \mathrm{Pr}\left(Y_{i}=K\right)=1-\sum_{k=1}^{K-1}\mathrm{Pr}\left(Y_{i}=k\right)=1-\sum_{k=1}^{K-1}\mathrm{Pr}\left(Y_{i}=K\right)e^{\beta_{k}\cdot X_{i}}\Rightarrow \mathrm{Pr}\left(Y_{i}=K\right)\\ =\frac{1}{1+\sum_{k=1}^{K-1}e^{\beta_{k}\cdot X_{i}}}. \end{array}$$

Respectively, we use the above to find the other possibilities [11, 18, 19].

(6) $$\begin{array}{c} \mathrm{Pr}\left(Y_{i}=1\right)=\frac{e^{\beta_{1}\cdot x_{i}}}{1+\sum_{k=1}^{K-1}e^{\beta_{k}\cdot X_{i}}},\\ \mathrm{Pr}\left(Y_{i}=2\right)=\frac{e^{\beta_{2}\cdot X_{i}}}{1+\sum_{k=1}^{K-1}e^{\beta_{k}\cdot X_{i}}},\\ \cdots \cdots \\ \mathrm{Pr}\left(Y_{i}=K-1\right)=\frac{e^{\beta_{K-1}\cdot X_{i}}}{1+\sum_{k=1}^{K-1}e^{\beta_{k}\cdot X_{i}}}. \end{array}$$

**Figure 1:**
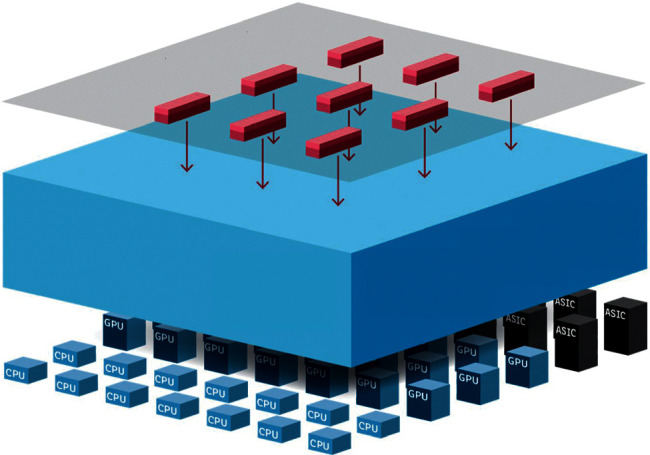
An abstract depiction of the proposed architecture.

The fact that we performed multiple regressions proves why we assumed the independence of irrelevant alternatives. Thus, the estimation of the desired distributed solution is feasible. An abstract illustration of the proposed architecture, based on how the schema can work in MDML, is shown in Figure 1.

By this logic, this model is a distributed machine learning technique as the proposed learning algorithm is implemented in multiple nodes to improve performance, increase accuracy, and distribute the input data of a learning model. This distributed nature of the proposed algorithm allows substantiated decisions to be made from large data sets [20, 21].

## 4. Use Case

For the modeling of the proposed system, a specialized ATA scenario was implemented, a threat that is one of the most critical risks of music content streaming applications today. Cybercriminals have developed a substantial criminal interest in the music ecosystem. The influx of visitors combined with the vast amounts of music content spent daily creates a new landscape of threats [22]. By this logic, they often utilize advanced techniques, even zero-day attacks, to steal credentials and generally launch ATA on music streaming platforms. The most common tactic is to use bot infrastructure to capture the accounts of unsuspecting users and exploit them for financial gain [23, 24].

Given the distributed nature of these applications and their use typically by mobile applications, this scenario implements a complete and compact DMLR cyber security system. The data in use are about an innovative clickstream dataset inspired by how phishing campaigns are detected in conjunction with credit card fraud detection techniques [24–28]. The features used are presented in detail in Table 1.

The DMLR presumes that the information is case-specific. For each scenario, each independent variable has a unique value. The model also implies that the independent variables can predict the dependent variable properly. There is no requirement for the independent variables to be statistically independent, as with other types of regression. However, coliteracy is regarded as relatively low because it becomes difficult to distinguish between the effects of several variables if the contrary is true [8, 16].

When used to model alternatives, the polynomial logit function relies on the independence of irrelevant alternatives (IIA), which is not always desirable. This implies that the likelihood of selecting one class over another is independent of the presence or absence of other irrelevant choices [29]. For example, if a bicycle is offered as an additional choice, the relative odds of choosing a car or bus to get to work do not vary. This enables the modeling of a set of *K* − 1 independent binary options as a collection of selected K alternatives. Each separate is chosen as a constant, and the other *K* − 1s are compared to it one by one. Although hypothesis IIA is a core hypothesis in rational choice theory, several psychological investigations reveal that people frequently breach this criterion when making decisions [30, 31].

When the polynomial logit is used to model options, it might produce too much confusion between the meaningful choices and between different alternatives in some instances. This is critical if the study's goal is to forecast how options would change if one of them disappeared. In comparable cases, other models, such as the nested logit or the polynomial probit, can be utilized because they enable the violation of the IIA.

As a log-linear model, binary accounting regression can be extended to multiple models. The logarithm of the separation function is obtained by modeling the logarithm of the probability of seeing a specific output with the linear classifier and a normalizing factor [32–35].

(7) $$\begin{array}{c} \ln\;\mathrm{Pr}\left(Y_{i}=1\right)=\beta_{1}\cdot {\text{X}}_{i}-\ln\;Z,\\ \ln\;\mathrm{Pr}\left(Y_{i}=2\right)=\beta_{2}\cdot {\text{X}}_{i}-\ln\;Z,\\ \;\cdots \\ \ln\;\mathrm{Pr}\left(Y_{i}=K\right)=\beta_{K},\\ \ln\;\mathrm{Pr}\left(Y_{i}=K\right)=\beta_{K}\cdot {\text{X}}_{i}-\ln\;Z. \end{array}$$

As in the binary case, we need an additional term −ln *Z* to ensure that all probabilities form a probability distribution so that they have a sum of 1.

(8) $$\begin{array}{c} \sum_{k=1}^{K}\mathrm{Pr}\left(Y_{i}=k\right)=1. \end{array}$$

We need to add a condition to ensure normalization, in addition to the usual multiplication, because we have taken the logarithm of the probabilities [36]. By increasing the power of each member of the equations, we convert the prosthetic part into a multiplier, and the probability becomes the measure of Gibbs [37].

(9) $$\begin{array}{c} \mathrm{Pr}\left(Y_{i}=1\right)=\frac{1}{Z}e^{\beta_{1}\cdot x_{i}},\\ \mathrm{Pr}\left(Y_{i}=2\right)=\frac{1}{Z}e^{\beta_{2}\cdot X_{i}},\\ \cdots \cdots \\ \mathrm{Pr}\left(Y_{i}=K\right)=\frac{1}{Z}e^{\beta_{K}\cdot X_{i}}. \end{array}$$

The quantity *Z* is known as the distribution partition function. We can compute the value of this function by applying the preceding constraint, which demands that all probabilities have a sum of 1.

(10) $$\begin{array}{c} 1=\sum_{k=1}^{K}\mathrm{Pr}\left(Y_{i}=k\right)=\sum_{k=1}^{K}\frac{1}{Z}e^{\beta_{k}\cdot x_{i}}=\frac{1}{Z}\sum_{k=1}^{K}e^{\beta_{k}\cdot x_{i}},\\ Z=\sum_{k=1}^{K}e^{\beta_{k}\cdot x_{i}}. \end{array}$$

The coefficient is a constant because it is not a function of *Y*_*i*_ which is the variable thanks to which the probability distribution is defined. However, it is not variable about the explanatory variables or unknown *β*_*k*_ coefficients of regression, which should be determined through an optimization process. The final equations of probability are [38–40]

(11) $$\begin{array}{c} \mathrm{Pr}\left(Y_{i}=1\right)=\frac{e^{\beta_{1}\cdot X_{i}}}{\sum_{k=1}^{K}e^{\beta_{k}\cdot x_{i}}},\\ \mathrm{Pr}\left(Y_{i}=2\right)=\frac{e^{\beta_{2}\cdot X_{i}}}{\sum_{k=1}^{K}e^{\beta_{k}\cdot x_{i}}},\\ \cdots \\ \mathrm{Pr}\left(Y_{i}=K\right)=\frac{e^{\beta_{K}\cdot X_{i}}}{\sum_{k=1}^{K}e^{\beta_{k}\cdot x_{i}}},\\ \mathrm{Pr}\left(Y_{i}=c\right)=\frac{e^{\beta_{c}\cdot X_{i}}}{\sum_{k=1}^{K}e^{\beta_{k}\cdot X_{i}}},\\ \text{softmax}\left(k,x_{1},\ldots ,x_{n}\right)=\frac{e^{x_{k}}}{\sum_{i=1}^{n}e^{x_{i}}} \end{array}$$

is called a softmax function. The reason is that the exposure of the variables (*x*_*1*_ ,   …  ,  *x*_*n*_) magnifies the differences between them. As a result, softmax (*k,x*_*1*_ ,   …  ,  *x*_*n*_) [41, 42]will return a value close to 0 when *x*_*k*_ is much less than the maximum of all values and will return 1 when applied to the maximum value, unless very close to the next highest price.

This function may build a weighted average that acts like a smooth function (and can be easily separated) and values the index function.

(12) $$\begin{array}{c} f\left(k\right)=\left\{\begin{array}{c} 1,k=\arg\max\left(x_{1},\ldots ,x_{n}\right)\\ 0,\text{otherwise} \end{array}\right\}. \end{array}$$

Thus, we can write the probability equations as follows:

(13) $$\begin{array}{c} \mathrm{Pr}\left(Y_{i}=c\right)=\text{soft }\max\left(c,\beta_{1}\cdot X_{i},\ldots ,\beta_{K}\cdot X_{i}\right). \end{array}$$

This function is the equivalent of the accounting function in binary accounting regression.

In general, there are only *k* − 1 individually differentiable probabilities, and therefore, there are *k* − 1 separately distinct coefficient vectors. One way to look at this is to see that if we add a constant vector to all factor vectors, then the equations are identical.

(14) $$\begin{array}{c} \frac{e^{\left(\beta_{c}+C\right)\cdot x_{i}}}{\sum_{k=1}^{K}e^{\left(\beta_{k}+C\right)\cdot x_{i}}}=\frac{e^{\beta_{c}\cdot x_{i}}e^{C\cdot x_{i}}}{\sum_{k=1}^{K}e^{\beta_{k}\cdot x_{i}}e^{C\cdot x_{i}}}=\frac{e^{C\cdot x_{i}}e^{\beta_{c}\cdot x_{i}}}{e^{C\cdot x_{i}}\sum_{k=1}^{K}e^{\beta_{k}\cdot x_{i}}}=\frac{e^{\beta_{c}\cdot x_{i}}}{\sum_{k=1}^{K}e^{\beta_{k}\cdot x_{i}}}. \end{array}$$

As a result, it is common to set *C*=−*βK* (or someone other than the coefficient vector). Essentially, we set the variable so that one of the vectors becomes 0, and the remaining vectors are changed into the difference between these vectors and the vectors we chose. The coefficients are transformed mathematically as follows [43]:

(15) $$\begin{array}{c} \beta_{1}^{\text{'}}=\beta_{1}-\beta_{K},\\ \cdots \cdots \\ \beta_{K-1}^{\text{'}}=\beta_{K-1}-\beta_{K},\\ \beta_{K}^{\text{'}}=0. \end{array}$$

This leads to the following equations [13, 35]:

(16) $$\begin{array}{c} \mathrm{Pr}\left(Y_{i}=1\right)=\frac{e^{\beta_{1}^{\text{'}}\cdot x_{i}}}{1+\sum_{k=1}^{K-1}e^{\beta_{k}^{\text{'}}\cdot x_{i}}},\\ \cdots \\ \mathrm{Pr}\left(Y_{i}=K-1\right)=\frac{e^{\beta_{K-1}^{\text{'}}\cdot x_{i}}}{1+\sum_{k=1}^{K-1}e^{\beta_{k}^{\text{'}}\cdot x_{i}}},\\ \mathrm{Pr}\left(Y_{i}=K\right)=\frac{1}{1+\sum_{k=1}^{K-1}e^{\beta_{k}^{\text{'}}\cdot x_{i}}}. \end{array}$$

The distributed configuration, which is based on the configuration server and is adopted here, was acquired in 8 different contexts, and the results of the process are presented in Table 2.

The proposed method attempts to approach the amount of Pr(*Y*_*i*_=1) by performing the sampling for a few repetitions. Binary regression units are initialized to a sample of actual data and perform *N* iterations, taking some data due to the model contribution [10, 44, 45]. Essentially, the method undertakes to give lower importance to the real data and much higher energy in the cases where a marginal distribution comes from, thus helping the model to approach the actual data distribution and determine the nature of the ATA.

In conclusion, the proposed architecture can accurately learn *N* samples from *N* − 1 models, while its learning speed can be even thousands of times faster than conventional methods. It is noteworthy that all *βk* coefficient vectors are uniquely recognizable. This has to do with the fact that all the odds must be added to 1, making one of them wholly decided while all the others are unknown. This can be translated, in combination with the very stable ability to predict, as the absence of contradictions between the models that act uniformly and respectively the existence of complete information and information about all the elements that make up the problem, so that the purpose of the decision comes from the choice of the optimal solution that maximizes the objective function and at the same time satisfies specific criteria.

## 5. Conclusions

In this work, we proposed an innovative MDML system, which significantly reduces the workload of servers in distributed environments and undertakes to perform machine learning tasks on large-scale data. Specifically, a distributed logit polynomial function model was proposed, which is used to model options in distributed binary regression accounting units. It is a highly efficient and low-demand distributed machine learning system tested to solve a highly demanding cybersecurity problem associated with distributed music repositories. Given the massive popularity of digital music repositories, targeted ATA attacks are carried out with malicious intent, such as stealing and removing personal information from user accounts.

As it turned out experimentally, the proposed system managed to categorize with great success the elements that lead to ATA attacks patterns. The high accuracy combined with the generalization of the results of the extensive tests indicates that this system is suitable for distributed environments and for solving highly complex problems. It is essential to say that the proposed standard had very stable predictability (0.9635 <F-score <0.9818), which proves the great coherence of the models that act uniformly in recognition of standards and respectively the existence of complete information and information for all elements that make up the problem, even if they come from a distributed environment.

In the future, the proposed method can be extended by developing a mobile application that will work in an even more efficient and flexible federated style, allowing several users to contribute to the model training process. By merging unsupervised and supervised learning methodologies, the distributed training algorithm may be optimized and operated at an abstract level to handle more complex and multidimensional data in more extensive data sets. Furthermore, some bioinspired optimization approaches, such as the particle optimization methodology, can be utilized to develop solutions that maximize or reduce some study parameters, such as the cost function. Finally, it would be fascinating to use the polynomial logit function to create a distributed neural network in which kernel technology would be used to address issues with nonlinearity but time continuities.

## Figures and Tables

**Table 1:** Features of dataset.

ID	Feature name	Type	ID	Feature name	Type
1	Age_Of_Domain	{1,−1}	2	Having_Ip_Address	{−1,1}
3	HTTPS_Token	{−1,1}	4	URL_Length	{1,0,−1}
5	Shortining_Service	{1,−1}	6	Having_At_Symbol	{1,−1}
7	Double_Slash_Redirecting	{−1,1}	8	Prefix_Suffix	{1,1}
9	Having_Sub_Domain	{−1,0,1}	10	Sslfinal_State	{−1,0,1}
11	Domain_Registeration_Length	{−1,1}	12	Favicon	{−1,1}
13	Port	{−1,1}	14	Request_Url	{−1,1}
15	URL_Of_Anchor	{−1,0,1}	16	Links_In_Tags	{−1,0,1}
17	Sfh	{−1,0,1}	18	Submitting_To_e-mail	{−1,1}
19	Abnormal_URL	{−1,1}	20	Redirect	{−1,1}
21	On_Mouseover	{−1,1}	22	Rightclick	{−1,1}
23	Popupwidnow	{−1,1}	24	Iframe	{−1,1}
25	Dnsrecord	{−1,1}	26	Web_Traffic	{−1,0,1}
27	Page_Rank	{−1,1}	28	Google_Index	{−1,1}
29	Links_Pointing_To_Page	{−1,0,1}	30	Statistical_Report	{−1,1}
31	Char_Freq_;	Real	32	Char_Freq_(	REAL
33	Char_Freq_[	Real	34	Char_Freq_!	REAL
35	Char_Freq_$	Real	36	Char_Freq_#	REAL
37	“Is_Host_Login”	{−1,1}	38	“Is_Guest_Login”	{−1,1}
39	“Num_Failed_Logins”	Real	40	“Logged_In”	{−1,1}
41	“Root_Shell”	Real	42	“Su_Attempted”	REAL
43	“Num_Root”	Real	44	Credit_Amount	REAL
45	Credit_History	“No credits,” “All paid,” “Existing paid,” “Delayed paid,” “Critical”	46	Class	Attack/Normal

**Table 2:** Model performance.

ID	Model Part	ROC Curve	F-score	Recall	Precision
1	Part-1	0.9777	0.9741	0.9770	0.9770
2	Part-2	0.9789	0.9735	0.9745	0.9776
3	Part-3	0.9663	0.9621	0.9610	0.9650
4	Part-4	0.9839	0.9818	0.9885	0.9861
5	Part-5	0.9747	0.9761	0.9735	0.9714
6	Part-6	0.9630	0.9635	0.9677	0.9660
7	Part-7	0.9786	0.9743	0.9750	0.9779
**Summary**		**0.9741**	**0.9719**	**0.9734**	**0.9738**

## Data Availability

The data used in this study are available from the author upon request.
